# Mouse and Human Monoclonal Antibodies Protect against Infection by Multiple Genotypes of Japanese Encephalitis Virus

**DOI:** 10.1128/mBio.00008-18

**Published:** 2018-02-27

**Authors:** Estefania Fernandez, Nurgun Kose, Melissa A. Edeling, Jagat Adhikari, Gopal Sapparapu, Susana M. Lazarte, Christopher A. Nelson, Jennifer Govero, Michael L. Gross, Daved H. Fremont, James E. Crowe, Michael S. Diamond

**Affiliations:** a Department of Pathology and Immunology, Washington University School of Medicine, Saint Louis, Missouri, USA; b Department of Pediatrics, Vanderbilt University Medical Center, Nashville, Tennessee, USA; c The Vanderbilt Vaccine Center, Vanderbilt University Medical Center, Nashville, Tennessee, USA; d Department of Chemistry, Washington University in St. Louis, Saint Louis, Missouri, USA; e Department of Medicine, Washington University School of Medicine, Saint Louis, Missouri, USA; f Department of Medicine, University of Texas Southwestern Medical School, Dallas, Texas, USA; g Andrew M. and Jane M. Bursky Center for Human Immunology and Immunotherapy Programs, Washington University School of Medicine, Saint Louis, Missouri, USA; h Department of Molecular Microbiology, Washington University School of Medicine, Saint Louis, Missouri, USA; i Department of Biochemistry and Molecular Biophysics, Washington University School of Medicine, Saint Louis, Missouri, USA; Sanofi (United States)

**Keywords:** antibody, flavivirus, immunotherapy, pathogenesis, virology

## Abstract

Japanese encephalitis virus (JEV) remains a leading cause of viral encephalitis worldwide. Although JEV-specific antibodies have been described, an assessment of their ability to neutralize multiple genotypes of JEV has been limited. Here, we describe the development of a panel of mouse and human neutralizing monoclonal antibodies (MAbs) that inhibit infection in cell culture of four different JEV genotypes tested. Mechanism-of-action studies showed that many of these MAbs inhibited infection at a postattachment step, including blockade of virus fusion. Mapping studies using site-directed mutagenesis and hydrogen-deuterium exchange with mass spectrometry revealed that the lateral ridge on domain III of the envelope protein was a primary recognition epitope for our panel of strongly neutralizing MAbs. Therapeutic studies in mice demonstrated protection against lethality caused by genotype I and III strains when MAbs were administered as a single dose even 5 days after infection. This information may inform the development of vaccines and therapeutic antibodies as emerging strains and genotypic shifts become more prevalent.

## IMPORTANCE

Although Japanese encephalitis virus (JEV) is a vaccine-preventable cause of viral encephalitis, the inactivated and live attenuated platforms available are derived from strains belonging to a single genotype (GIII) due to its historical prevalence in areas of JEV epidemics. Related to this, studies with vaccines and antibodies have focused on assessing the *in vitro* and *in vivo* protective responses to homologous or heterologous GIII strains. An epidemiological shift in JEV genotype distribution warrants the induction of broadly neutralizing antibody responses that inhibit infection of multiple JEV genotypes. Here, we generated a panel of mouse and human neutralizing monoclonal antibodies and evaluated their inhibitory activity, epitope location, and capacity for protection against multiple JEV genotypes in mice.

## INTRODUCTION

Despite the existence of inactivated and live attenuated vaccine platforms, Japanese encephalitis virus (JEV) remains a primary cause of viral encephalitis. It is particularly prevalent in Asia, with approximately 68,000 clinical cases (1, 2) and an estimated 10,000 to 15,000 deaths per year (1). JEV circulation is endemic in southern tropical and subtropical areas (e.g., Australia, Indonesia, and Singapore), with epidemics occurring in northern temperate regions (e.g., Japan, Bhutan, and Nepal) (3, 4). JEV is transmitted primarily by the *Culex tritaeniorhynchus* mosquito and is maintained in an enzootic cycle with pigs and wading birds. In contrast, humans are infected as incidental dead-end hosts (5, 6). The high incidence of JEV in rural areas has been attributed to the presence of open water sources, the preferred breeding grounds for *Culex* mosquitoes (7).

Approximately 5 to 15 days after mosquito inoculation of JEV, a nonspecific febrile illness develops, characterized by malaise, headache, and general discomfort (2). Symptomatic JEV infection is observed most commonly in children in areas of endemicity, children and adults in areas with JEV epidemics, and travelers to areas of endemicity and epidemics (3, 8). Severe clinical JEV disease occurs in about 1% of infected humans, with progression to encephalitis, seizures, or neurological deficits (9, 10). Beyond death, which occurs in 20 to 30% of clinical cases, severe long-term complications include paralysis, dystonia, and cognitive deficits (10–12).

JEV is a flavivirus of the *Flaviviridae* family and is related to other viruses that cause human disease, including Zika (ZIKV), West Nile (WNV), dengue (DENV), tick-borne encephalitis (TBEV), and yellow fever (YFV) viruses. JEV is an ~50-nm enveloped, positive-stranded RNA virus with an ~11-kb genome flanked by 5′ and 3′ untranslated regions. The genome encodes a single open reading frame that is co- and posttranslationally cleaved by viral and host proteases into three structural proteins (capsid [C], premembrane [prM], and envelope [E]) and seven nonstructural proteins. The E protein is necessary for virus binding, entry, and fusion in host cells (13) and the ectodomain is divided into three domains: domain I (E-DI) is the central β-barrel domain, domain II (E-DII) is an extended dimerization domain with a distal hydrophobic fusion loop (FL), and domain III (E-DIII) is an immunoglobulin-like fold (14). Structural analysis of the JEV E protein shows a smaller dimer interface with increased contacts at the E-DI-DIII pocket compared to those of related flaviviruses (15).

Although most phylogenetic analyses define four JEV genotypes based on sequence variation of the E protein, multiple strains belonging to a fifth genotype were recently identified in Malaysia and South Korea (16–18). The genotypes cluster within particular geographic distributions: for example, genotype I (GI) and GIII strains are more common in temperate regions, whereas GII and GIV strains are more common in tropical climates (19–21). GIII has been the predominant genotype historically, and as such, existing vaccines against JEV are derived from prototypical GIII strains such as JEV-Nakayama and JEV-SA14 (21). Recent reports have noted a substantial increase in GI infections in Asian countries, including China and Japan (22, 23).

The humoral response to JEV, like that of other flaviviruses, is considered necessary for limiting infection, and neutralizing antibody titers often serve as a correlate of protection (24). Indeed, JEV type-specific mouse monoclonal antibodies (MAbs) with protective activity (e.g., E3.3) have been identified and were derived against GIII strains (25–28). Moreover, a humanized MAb (B2) that was derived from a chimpanzee immunized with JE-VAX also protected mice against JEV-Nakayama, a strain of the homologous JEV genotype (GIII) (29). Other neutralizing MAbs (e.g., 2H4 and 2F2) in goat and monkey models of infection (30) protected against JEV strains from the homologous genotype to which they were raised. Notwithstanding these data, no study has comprehensively profiled the inhibitory activity of anti-JEV MAbs against multiple genotypes *in vitro* and *in vivo*, and no fully human anti-JEV MAbs have been described. The shift in prevalence from GIII to GI may require a different antibody repertoire for protection against infection and thus has implications for the efficacy of existing vaccines that were derived from GIII strains.

Here, we generated a panel of mouse and human MAbs against JEV after immunizing mice and humans with a GIII vaccine strain (JEV-SA14-14-2) or mice with pathogenic GII and GIII strains of JEV. Six of the mouse MAbs (JEV-31, JEV-106, JEV-128, JEV-131, JEV-143, and JEV-169) neutralized infection of strains representative of the four JEV genotypes (GI, GII, GIII, and GIV) that we tested to various degrees. Site-directed mutagenesis and hydrogen-deuterium exchange mass spectrometry (HDX-MS) mapping data identified sites within E-DI (JEV-169), E-DIII (JEV-31, JEV-106, JEV-128, JEV-131, JEV-143, and hJEV-69), and additional regions of the E ectodomain (JEV-117 and hJEV-75) as key epitopes for neutralization. Passive transfer studies in lethal JEV challenge mouse models showed protective efficacy for some mouse and human MAbs even when administered up to 5 days after GI or GIII infection. These data may be relevant for the development of antibody-based therapeutics or anti-JEV vaccines with broader protective activity, which may be important as the predominant genotypes shift over time.

## RESULTS

### Anti-JEV MAbs.

We generated a panel of neutralizing murine MAbs against JEV to begin to address the impact of shifting genotype epidemiology on antibody-mediated protection. We inoculated and boosted adult C57BL/6 mice deficient for interferon (IFN) regulatory factor 3 (*Irf3*^*−/−*^) with 10^2^ focus-forming units (FFU) of a vaccine strain of JEV (JEV-SA14-14-2). Additionally, we inoculated *Irf7*^−/−^ mice with JEV-Nakayama (GIII), boosted with JEV-Bennett (GII), and administered a final intravenous boost with JEV-Nakayama before splenocyte-myeloma cell fusion. We immunized *Irf3*^*−/−*^ and *Irf7*^*−/−*^ rather than wild-type (WT) mice, as JEV replicated to higher titers and induced stronger neutralizing antibody responses in these animals (data not shown). We screened ~3,800 hybridoma supernatants from five independent fusions for binding to JEV-infected cells by flow cytometry and direct virus binding by enzyme-linked immunosorbent assay (ELISA) and cloned 13 JEV MAbs by limiting dilution for further characterization. Using a single-endpoint neutralization assay, we identified 8 MAbs with >95% neutralizing activity against infection of JEV-SA14-14-2 in Vero cells (data not shown).

We then tested these mouse MAbs for their ability to bind recombinant JEV E ectodomain, JEV E-DI, JEV E-DIII, WNV E ectodomain, or ZIKV E ectodomain by ELISA (Table 1). JEV-169 bound E-DI, and the remaining MAbs recognized E-DIII, with the exception of JEV-117, which recognized JEV E ectodomain but not the domain fragments. JEV-31 and JEV-117 showed cross-reactivity to WNV E protein, whereas JEV-143 cross-reacted with ZIKV E protein.

**TABLE 1:** Binding and neutralization of inhibitory anti-JEV MAbs

Mab	Isotype^a^	Domain^a^	Cross-reactivity^a^	FRNT_50_ (ng/ml)^b^						
				GI		GII, Bennett	GIII			GIV, JKT 7887
				2372/79	MAR 859		Nakayama	SA14	SA14-14-2	
Mouse										
JEV-27	IgG2c	DIII	N	4,830	4,053	3,846	2,332	1,441	1,779	2,433
JEV-31	IgG2c	DIII	W	365	272	241	223	94	84	211
JEV-106	IgG2c	DIII	N	449	500	548	334	147	199	270
JEV-117	IgG2c	N	W	>10,000	>10,000	>10,000	>10,000	>10,000	11	>10,000
JEV-128	IgG2c	DIII	N	1,629	561	276	267	189	102	555
JEV-131	IgG2c	DIII	N	509	336	263	409	207	95	815
JEV-143	IgG2c	DIII	Z	435	405	358	346	368	379	818
JEV-169	IgG2c	DI	N	69	80	88	112	148	49	315

Human										
hJEV-11	hIgG1, κ	DIII	W	5,445	1,509	4,116	>10,000	4,528	2,226	>10,000
hJEV-69	hIgG1, κ	DIII	N	1,102	335	524	2,444	475	211	3,111
hJEV-75	hIgG1, λ	N	N	457	228	388	294	414	9	>10,000
hJEV-80	hIgG1, λ	DIII	W	3,371	1,117	1,036	>10,000	857	1,007	7,733

a Immunoglobulin isotype, domain specificity, and cross-reactivity to WNV (W) and ZIKV (Z) were determined by ELISA. “N” indicates no binding to either WNV (W) or ZIKV (Z) recombinant E protein or JEV E protein domains.

b Purified MAb was incubated with 10^2^ FFU of the indicated JEV strain of genotypes GI to GIV for 1 h at 37°C. Fifty percent FRNT (FRNT_50_) values were determined by nonlinear regression. Results are the average from three independent experiments performed in triplicate.

**FIG 1:**
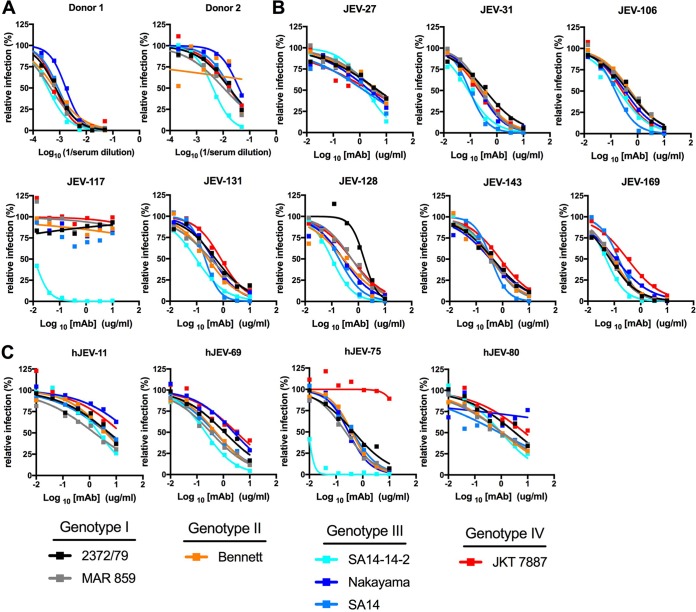
Neutralization activity of anti-JEV MAbs. (A) Serum samples from humans previously immunized against JEV with an inactivated virion vaccine were tested against a panel of JEV strains (2372/79 [GI], MAR 859 [GI], Bennett [GII], SA14 [GIII], SA14-14-2 [GIII], Nakayama [GIII], and JKT 7887 [GIV]) by focus-forming assay (FFA) for neutralization activity. Serial serum dilutions were incubated with 10^2^ FFU for 1 h at 37°C, and Vero cells were subsequently infected and stained. (B) Neutralization curves of eight mouse anti-JEV MAbs (JEV-27, JEV-31, JEV-106, JEV-117, JEV-131, JEV-128, JEV-143, and JEV-169) against the indicated strains. (C) Neutralization curves of human-derived anti-JEV MAbs (hJEV-11, hJEV-69, hJEV-75, and hJEV-80) against the indicated strains. All data are representative of three independent experiments performed in triplicate.

To generate human MAbs against JEV, we screened neutralization profiles from donors immunized with a two-dose regimen of a commercially available inactivated JEV vaccine, IXIARO, that was based on a genotype III strain (Fig. 1A). We obtained hybridoma supernatants derived from donors that bound to JEV-SA14-14-2, determined the single-endpoint neutralization titer (data not shown), and cloned 4 anti-JEV MAbs. Three of the human MAbs bound to E-DIII, whereas hJEV-75 bound to the E ectodomain but not to E-DI or E-DIII (Table 1). hJEV-11 and hJEV-80 cross-reacted with WNV E protein, whereas hJEV-69 and hJEV-75 appeared specific to JEV and did not bind either WNV or ZIKV E proteins.

### Breadth of neutralization of MAbs.

We performed focus reduction neutralization tests (FRNTs) on Vero cells to assess the inhibitory capacity of our anti-JEV MAbs against the vaccine strain, JEV-SA14-14-2, and available prototype strains representative of multiple genotypes. We did not test a representative genotype V strain of JEV, as one was not available from the World Arbovirus Reference Collection. We determined the MAb concentration that reduced the number of foci of infection by 50% (50% effective concentration [EC_50_]) (Fig. 1B and C; Table 1). JEV-31 and JEV-169 had the strongest neutralization activity against the four genotypes tested (GI, GII, GIII, and GIV), with EC_50_ values between 84 and 365 ng/ml and 49 and 315 ng/ml, respectively. JEV-106, JEV-128, JEV-131, and JEV-143 had intermediate neutralizing activity, with EC_50_ values between 147 and 548 ng/ml, 102 and 1,629 ng/ml, 95 and 509 ng/ml, and 346 and 818 ng/ml, respectively, against strains of the four genotypes. As expected, the JEV-SA14-14-2 vaccine and JEV-SA14 parental strain were neutralized to similar levels by most MAbs, with the exception of JEV-117, which showed a remarkable ~1,000-fold shift in EC_50_ values. In general, JEV-27 and JEV-117 had the weakest neutralizing activity, with EC_50_ values between 1,441 and 4,830 ng/ml and >10,000 ng/ml, respectively.

We identified four human MAbs with neutralizing activity against JEV-SA14-14-2, which we characterized in greater detail. hJEV-11 and hJEV-80 exhibited relatively weak neutralizing activity (1,509 to 10,000 ng/ml and 857 to 10,000 ng/ml, respectively) against the other strains tested (Fig. 1C; Table 1). In comparison, hJEV-69 and hJEV-75 inhibited infection of multiple JEV strains more potently. hJEV-69 had greater activity against the GI strains (2372/79 and MAR 859; EC_50_, 335 to 1,102 ng/ml) than against the GIV strain (JKT 7887; EC_50_, 3,111 ng/ml), whereas hJEV-75 had the strongest neutralizing activity against GI, GII, and GIII strains (EC_50_, 9 to 457 ng/ml) but did not inhibit the GIV strain (JKT 7887; EC_50_, >10,000 ng/ml). Overall, the mouse-derived MAbs had greater breadth of neutralization against multiple genotypes of JEV than the human-derived MAbs. This finding could reflect the different immunogens used (live versus inactivated viruses for mice or humans, respectively), species-specific differences in the antibody repertoire, or the limited size of the panel of MAbs that we obtained.

### Mechanism of neutralization.

**FIG 2:**
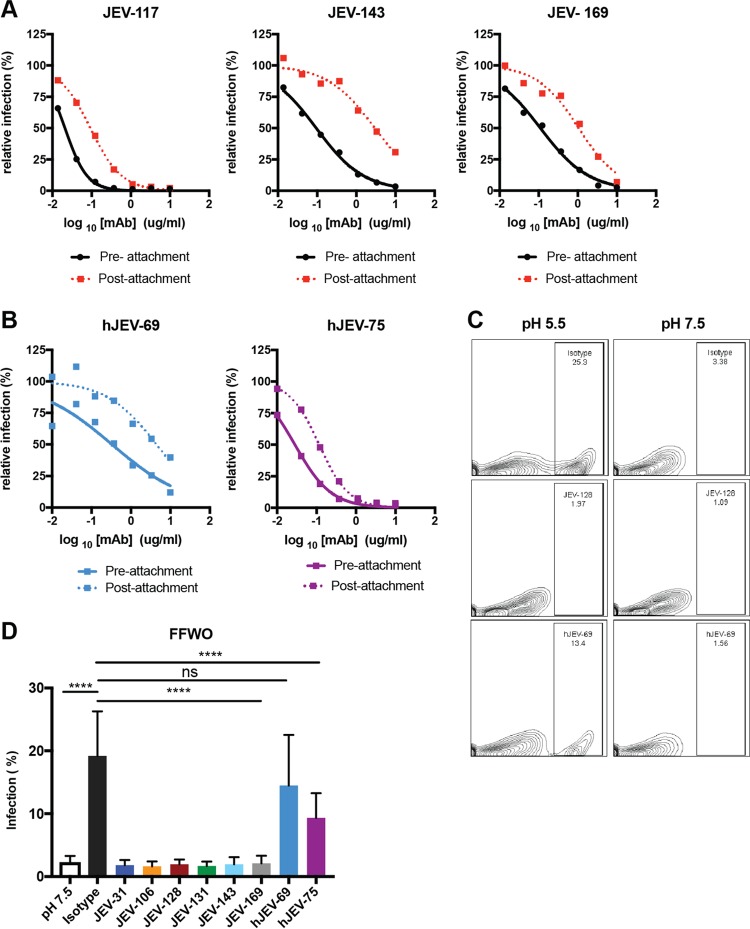
Mechanism of neutralization by anti-JEV MAbs. (A and B) The preattachment inhibition assay (solid lines) was performed by incubating 10^2^ FFU of JEV-SA14-14-2 with serial dilutions of MAbs starting at 10 µg/ml for 1 h at 4°C before addition to prechilled Vero cells at 4°C and subsequently following the FFA protocol. The postattachment assay (dashed lines) was performed by adding 10^2^ FFU of JEV-SA14-14-2 to cells for 1 h at 4°C. After extensive washing to remove unbound virus, serial dilutions of MAbs were added, starting at 10 µg/ml, and incubated for 1 h at 4°C, and the FFA then was completed at 37°C. Data are representative of three experiments performed in triplicate. (C) The fusion-from-without (FFWO) assay was performed after incubating Vero cells at 4°C with JEV-SA14 (MOI of 50) for 2 h. For these experiments, we used JEV-SA14 instead of JEV-SA14-14-2 because it could be grown to a higher titer. Cells were washed extensively, and the indicated MAbs were added for 30 min. Plasma membrane fusion was induced by exposing the cells briefly (~7 min) to an acidic pH buffer. After pH normalization, cells were incubated with 10 nM concanamycin for 24 h to inhibit infection via the endosomal pathway and collected, fixed, permeabilized, and stained for E protein expression. The treatment and percentage of positive cells are shown. (D) The data are pooled from three independent experiments, each performed in triplicate, with error bars (standard deviation) and were analyzed using one-way ANOVA with Dunnett’s multiple comparisons to the isotype control condition. ****, *P* < 0.0001; ns, not significant.

Antibody neutralization of flaviviruses can occur by inhibiting attachment, internalization, and/or fusion (31). To determine how the neutralizing MAbs inhibited infection in cell culture, we performed pre- and postattachment neutralization assays (32–34). MAbs were incubated with JEV-SA14-14-2 before or after virus binding to cells, and infection was measured by FRNT (32–34). As expected, all MAbs efficiently neutralized infection when premixed with virus (Fig. 2A; see Fig. S1 [solid lines] in the supplemental material). All mouse MAbs also inhibited JEV infection when added after virus adsorption to the cell surface, although to a lesser extent, suggesting that at least part of their blocking activity was at a post-attachment step (Fig. 2A; Fig. S1, dashed lines). Similarly, hJEV-69 and hJEV-75 neutralized in both pre- and post-attachment assays (Fig. 2B).

10.1128/mBio.00008-18.1 FIG S1  Mechanism of neutralization by JEV neutralizing MAbs. Shown are the pre- and postattachment assays for mouse MAbs (JEV-31, JEV-106, JEV-128, and JEV-131) against JEV-SA14-14-2, as described in Fig. 2. Data are representative of three experiments, each performed in triplicate. Download FIG S1, TIF file, 0.4 MB. Copyright © 2018 Fernandez et al. 2018 Fernandez et al. This content is distributed under the terms of the Creative Commons Attribution 4.0 International license.

We next determined whether the neutralizing mouse and human MAbs could block fusion by adapting a virus fusion from without (FFWO) assay at the plasma membrane (32, 33). JEV-SA14 was adsorbed to a monolayer of Vero cells on ice and subsequently incubated with the MAbs. Fusion at the plasma membrane was induced by brief exposure to low-pH-buffered medium at 37°C. After washing, cells were incubated overnight in the presence of 10 nM concanamycin A1 to prevent canonical endosomal fusion and allow viral replication. As described for other flaviviruses (33), in the absence of MAb treatment, ~20% of cells produced viral antigen that was measurable by flow cytometry; in contrast, minimal viral antigen (~2 to 3% of cells) was detected when fusion was induced under neutral-pH conditions (Fig. 2C and D). All neutralizing mouse MAbs tested inhibited plasma membrane fusion under acidic conditions and subsequent viral antigen expression. In contrast, hJEV-69 and hJEV-75 inhibited fusion at the plasma membrane less efficiently (Fig. 2C and D).

### Epitope mapping.

To begin to assess the basis for differential inhibition by the neutralizing MAbs, we mapped their epitopes. We defined key peptide regions and amino acid residues required for MAb binding by using both hydrogen-deuterium exchange mass spectrometry (HDX-MS) (35) and alanine-scanning site-directed mutagenesis (36) of the E protein of JEV-SA14-14-2.

### (i) HDX-MS.

**FIG 3:**
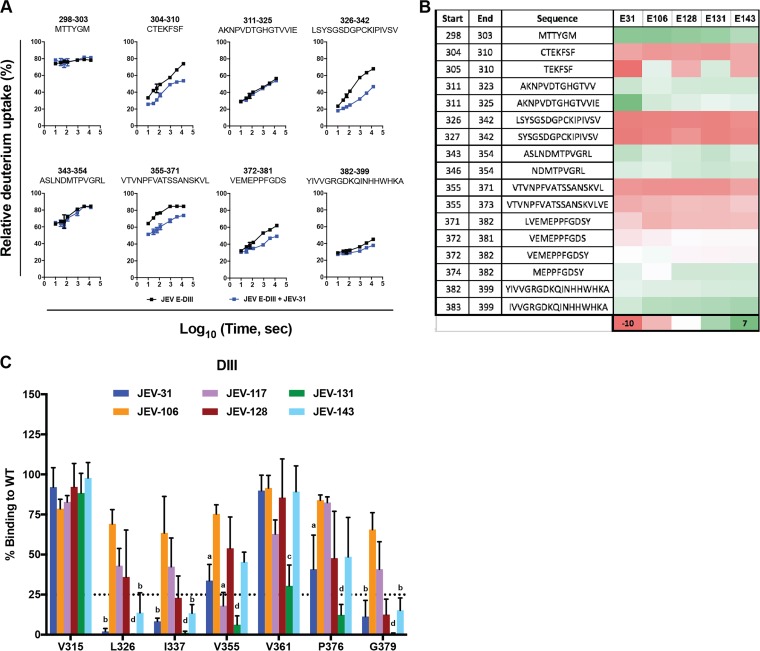
Epitope mapping by hydrogen-deuterium exchange and alanine-scanning mutagenesis. (A) Representative HDX kinetic plots for eight different peptides spanning JEV E-DIII in the presence (blue lines) or absence (black lines) of JEV-31. Regions showing reduced rates or extents of exchange are considered to contain the binding epitopes. All experiments were performed in duplicate, and data are representative of two independent experiments. (B) Heat map depicting the average difference of deuterium incorporation between E-DIII alone and the corresponding E-DIII-MAb complex states across all seven time points (ΔD%). Negative values of ΔD% indicate less deuterium incorporation in the DIII-MAb state. The regions with significant protection are shown in red. Peptides with no or little change in deuterium uptake are indicated by white and green. (C) Representative alanine-scanning mutagenesis. 293T cells were transfected with 1 µg of the indicated plasmid and incubated overnight prior to fixation, permeabilization, and staining with JEV-31, JEV-106, JEV-117, JEV-128, JEV-131, and JEV-143. Loss of binding was detected by flow cytometry. Data are representative of three independent experiments, with error bars (standard error of the mean [SEM]) and were analyzed by one-way ANOVA with Holm-Sidak’s multiple comparisons of each mutant compared to V315 for each MAb. Superscript letters indicate significance: a, *P* < 0.05; b, *P* < 0.01; c, *P* < 0.001; d, *P* < 0.0001.

As HDX-MS should show slower exchange at MAb binding sites (increased protection), we analyzed five mouse MAbs (JEV-31, JEV-106, JEV-128, JEV-131, and JEV-143) that engaged E-DIII. The MAbs were mixed in a 1:1 ratio with E-DIII, and HDX was performed for 10, 30, 60, 120, 900, 3,600, and 14,400 s. The quenching and protein digestion conditions were optimized to obtain 32 different peptides that spanned the 11-kDa JEV E-DIII protein (Fig. S2A). All five MAbs showed changes in deuterium uptake compared to unliganded E-DIII. Representative kinetic plots are shown for eight of the peptides spanning E-DIII in the presence of JEV-31 (Fig. 3A). The deuterium uptake studies showed that binding of JEV-31, JEV-106, JEV-128, JEV-131, and JEV-143 protected regions in the N-terminal region and A strand (residues 304 to 310), BC loop (residues 326 to 342), and DE loop (residues 355 to 371) of E-DIII (Fig. 3B; Fig. S2B), regions that correspond to the well-defined lateral ridge (LR) epitope (37) (E-DIII-LR).

10.1128/mBio.00008-18.2 FIG S2  Sequence coverage map of peptic digestion of JEV E-DIII. (A) A total of 32 peptides were identified with complete sequence coverage for JEV E-DIII. Each bar indicates a peptide identified by mass spectrometry. The colored bars represent the average deuterium uptake percentage (D%) for the duplicate analysis of seven exchange time points. (The warmer the color, the higher the deuterium uptake is.) The deuterium uptake percentages for the duplicate analyses are indicated inside the bars, along with the standard deviation and the charge states of the peptide in parentheses. (B) Comparison of the kinetics of HDX for eight different peptides covering the entire E-DIII in the absence (E-DIII alone, black lines) or presence (E-DIII plus MAbs) of various MAbs (colored lines). Each region (column) is represented by a peptic peptide, as measured by mass spectrometry. Each row represents a state bound with a MAb (JEV-106, orange; JEV-128, maroon; JEV-131, green; JEV-143, light blue); the antibody is listed on the left. Regions showing reduced rates of exchange for the sample of E-DIII with MAbs (non-black lines) are considered to contain the epitopes. Regions with no difference are examples of regions that do not contain the epitopes and can be viewed as controls. Download FIG S2, TIF file, 1.9 MB. Copyright © 2018 Fernandez et al. 2018 Fernandez et al. This content is distributed under the terms of the Creative Commons Attribution 4.0 International license.

### (ii) Alanine-scanning mutagenesis.

**FIG 4:**
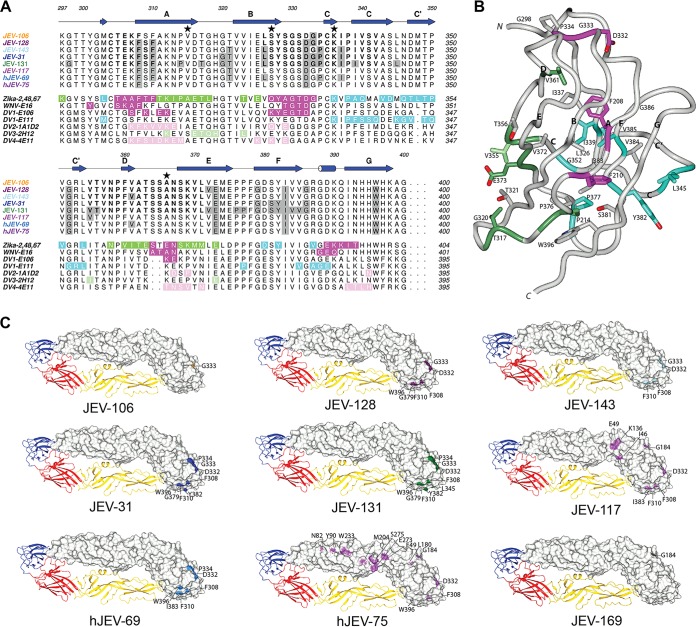
Structural representation of JEV E epitopes defined by alanine-scanning mutagenesis and HDX. (A) JEV E-DIII epitopes for each of the eight mouse and human JEV MAbs were defined by alanine-scanning mutagenesis (shaded gray boxes) and HDX (boldface letters). Genotypic differences from the JEV-SA14-14-2 strain (GIII) are highlighted by a star: V315 is A in the JEV-2372/79 (GI), JEV-MAR 859 (GI), JEV-Bennett (GII), and JEV-Nakayama (GIII) strains; S327 is T in the JEV-2372/79 (GI), JEV-MAR 859 (GI), and JEV-Bennett (GIII) strains; K336 is N in the JEV-2372/79 (GI) and JEV-MAR 859 (GI) strains; and A366 is S in the JEV-2372/79 (GI), JEV-MAR 859 (GI), and JEV-Bennett (GIII) strains. For comparison to the JEV E-DIII epitopes, immediately below we show the structurally defined E-DIII epitopes of ZIKV in complex with ZV-2 (green, ABDE epitope), ZV-48 (cyan, C-C') and ZV-67 (magenta, lateral ridge [LR]), WNV E16 (magenta, LR), DV1-E106 (magenta, LR), DV1-E111 (cyan, C-C′ loop), DV2-1A1D-2 (pink, A strand), DV3-2H12 (light green, AB loop), and DV4-4E11 (pink, A strand). (B) JEV E-DIII epitopes defined by alanine-scanning mutagenesis are depicted on the JEV E-DIII structure (based on the full-length JEV E structure, PDB accession no. 3P54). (C) JEV epitopes defined by alanine-scanning mutagenesis, HDX mapping, and surface exposure are shown in the context of the full-length JEV E structure.

The amino acid binding sites of neutralizing mouse and human anti-JEV MAbs also were mapped by alanine-scanning mutagenesis and mammalian cell expression (36) of the JEV prM-E protein. Residues in the E protein ectodomain were replaced with alanine with two exceptions: alanine residues were mutated to serine, and cysteines were not mutated to prevent protein misfolding (Table S1). We characterized a residue as critical for MAb binding if the mutation resulted in less than 25% binding compared to the wild-type protein (Fig. 3C and 4). We found that alanine substitution of certain amino acids (e.g., T321, D332, and I383), which correspond to sites in E-DIII-LR, caused loss of binding of most of the neutralizing murine and human MAbs tested, especially JEV-31, JEV-131, JEV-143, and hJEV-69 (Fig. 4A and B). JEV-131 showed a broader binding footprint, as loss of binding was observed for alanine substitution of additional residues, including G299, L345, P376, and V384. JEV-117 and hJEV-75 demonstrated loss of binding following mutations in other regions of the E ectodomain (Fig. 4C) that correspond to previously defined epitopes for related flaviviruses, including residues in the E-DI-DII-hinge region (K136 for JEV-117 and S275 for hJEV-75), E-DI-LR (L180 for hJEV-75), E-DII-hinge (E49), E-DII-LR (N82 for hJEV-75), and E-DII-central interface (W217 for hJEV-75) (15, 38). The loss of binding observed within E-DIII for alanine substitutions of residues F308 (JEV-117 and hJEV-75) and F310 (JEV-117) corresponds to sites within the previously described A-strand epitope (39) (Table S1). This pattern of mutagenesis and binding also correlates with the inability of JEV-117 and hJEV-75 to recognize isolated domains by ELISA (Table 1). JEV-169 demonstrated loss of binding with three different mutations in DI (L25, G184, and L285) and a single mutation in DII (M204), although these residues do not correspond to any published epitope. Because alanine substitutions can have only moderate structural differences compared to other residues, we also made charge substitutions in amino acids at different E-DIII epitopes, including the A strand (S309K, K312E, and H395K), DIII-LR (S331K, S364K, N367K, and K369E), C-C′ loop (T349K), and FG loop (R387E and D389K). Loss of binding in the E-DIII-LR epitope (S331K and S364K) but not in other E-DIII regions was observed for the murine MAbs JEV-31, JEV-106, JEV-128, JEV-131, and JEV-143 (Table S2). Unexpectedly, we did not observe loss of binding for hJEV-69, suggesting it may recognize E-DIII somewhat differently than the neutralizing MAbs of mouse origin.

10.1128/mBio.00008-18.4 TABLE S1  Alanine-scanning mutagenesis of JEV E ectodomain. Shown is the percentage of binding of each MAb to the respective mutant compared to MAb binding to the WT plasmid. Data are representative of 2 replicates for >25% binding and 3 replicates for <25% binding. ND, not determined. Download TABLE S1, XLSX file, 0.1 MB. Copyright © 2018 Fernandez et al. 2018 Fernandez et al. This content is distributed under the terms of the Creative Commons Attribution 4.0 International license.

10.1128/mBio.00008-18.5 TABLE S2  JEV E-DIII charge mutagenesis of epitope-specific residues. Shown is the percentage of binding of each MAb to the respective mutant compared to MAb binding to the WT plasmid. Data are representative of 2 replicates. ND, not determined. Download TABLE S2, XLSX file, 0.1 MB. Copyright © 2018 Fernandez et al. 2018 Fernandez et al. This content is distributed under the terms of the Creative Commons Attribution 4.0 International license.

### *In vivo* protection studies.

To evaluate whether neutralizing MAbs could protect against JEV infection *in vivo*, we developed challenge models of JEV-induced lethality in mice by using GIII (Nakayama) and GI (MAR 859 and 2372/79) strains. Once models were established, we treated 4- to 5-week-old male WT C57BL/6 mice on day −1 with a single 10-μg (0.5-mg/kg) prophylactic dose of seven different anti-JEV MAbs or an isotype-control MAb and then inoculated animals on day 0 with different pathogenic JEV strains.

### (i) Nakayama (GIII).

**FIG 5:**
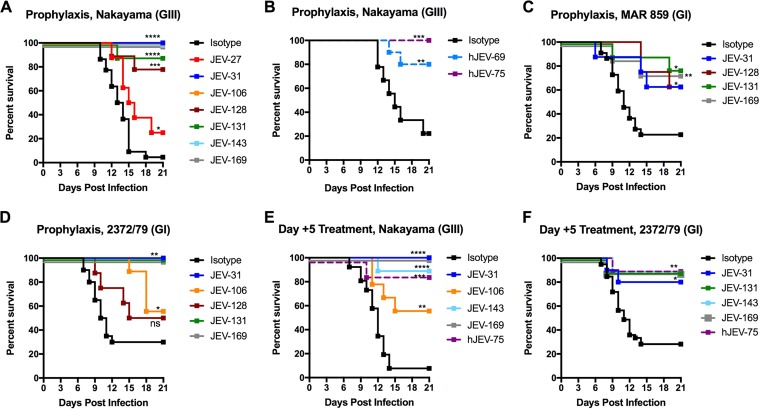
Protective efficacy of anti-JEV MAbs in mice. (A and B) Four- to 5-week-old male C57BL/6 mice were passively administered 10 µg of the indicated (A) mouse or (B) human MAb via intraperitoneal injection 1 day prior to inoculation with 10^2^ FFU of JEV-Nakayama via the subcutaneous route. JEV-31 (*n* = 9), JEV-106 (*n* = 8), JEV-143 (*n* = 8), and JEV-169 (*n* = 10) provided complete protection against lethality. JEV-27 (*n* = 8), JEV-128 (*n* = 9), and JEV-131 (*n* = 9) provided partial protection compared to the isotype control MAbs. (C and D) Three-week-old male C57BL/6 mice were passively administered 10 µg of the indicated MAb as described above 1 day prior to inoculation with 10^3^ FFU of (C) JEV-MAR 859 (JEV-31, *n* = 8; JEV-131, *n* = 9; JEV-169, *n* = 8) or (D) JEV-2372/79 (JEV-31, *n* = 9; JEV-106, *n* = 9; JEV-131, *n* = 9; JEV-169, *n* = 9). (E and F) Two hundred fifty micrograms of the indicated MAb was administered 5 days postinfection to (E) 4- to 5-week-old mice infected with 10^2^ FFU of JEV-Nakayama (JEV-31, *n* = 9; JEV-106, *n* = 9; JEV-143, *n* = 9; JEV-169, *n* = 9; hJEV-75, *n* = 8) or (F) 3-week-old mice infected with 10^3^ FFU of JEV-2372/79 (JEV-31, *n* = 10; JEV-131, *n* = 9; JEV-143, *n* = 9; JEV-169, *n* = 10; hJEV-75, *n* = 9). Data are pooled from at least two independent experiments. Survival was analyzed for each MAb compared to the isotype control MAb by the log rank test. *, *P* < 0.05; **, *P* < 0.01; ***, *P* < 0.001; ****, *P* < 0.0001; ns, not significant.

Whereas JEV-31, JEV-106, JEV-143, and JEV-169 protected all mice from lethal infection (Fig. 5A), JEV-27, JEV-128, and JEV-131 conferred partial (25 to 89%) protection. We also observed protection (60 to 80%) with similar doses of hJEV-69 and hJEV-75 (Fig. 5B).

### (ii) MAR 859 (GI).

JEV-31, JEV-128, JEV-131, and JEV-169 conferred partial protection, ranging from 40 to 55% (Fig. 5C).

### (iii) 2372/79 (GI).

JEV-31, JEV-131, and JEV-169 provided complete protection against lethality, whereas JEV-106 and JEV-128 provided more limited (25 to 30%) protection (Fig. 5D).

To define the therapeutic potential of our most protective MAbs, a single 250-μg (15-mg/kg) dose was administered to mice 5 days after infection (Fig. 5E and F). Whereas JEV-31 and JEV-169 completely protected against lethality induced by JEV-Nakayama (GIII), these MAbs showed more limited therapeutic activity against JEV-2372/79 (GI), as they protected 50 to 60% of mice, respectively. Administration of hJEV-75 at 5 days after infection also had significant protection against both JEV-Nakayama (GIII) and JEV-2372/79 (GI) strains. Overall, our data show that a single MAb that broadly neutralizes multiple JEV genotypes can provide therapeutic activity *in vivo* against multiple strains.

## DISCUSSION

We sought to identify murine and human MAbs that broadly neutralize infection of JEV strains corresponding to most genotypes. We inoculated mice with attenuated or infectious strains of JEV to generate a panel of eight anti-JEV MAbs and characterized them at the functional and structural levels. From our analyses, we identified three classes of antibodies based on neutralization profile, epitope binding, and *in vivo* efficacy. The two MAbs JEV-27 and JEV-117 had the weakest inhibitory profiles. Four MAbs (JEV-106, JEV-128, JEV-131, and JEV-143) had intermediate neutralization abilities, and two MAbs (JEV-31 and JEV-169) were strongly and broadly neutralizing. Binding analysis revealed two mouse MAbs (JEV-31 and JEV-117) that were cross-reactive with WNV. JEV-143 cross-reacted with ZIKV, and five other mouse MAbs (JEV-27, JEV-106, JEV-128, JEV-131, and JEV-169) appeared more type specific. JEV-31, which cross-reacted with WNV and was one of the most strongly neutralizing MAbs in our panel, recognized an epitope in the E-DIII-LR. A single JEV-specific neutralizing murine MAb, JEV-169, mapped to E-DI. We also generated the first human MAbs for JEV isolated from B cells of recipients of a chemically inactivated JEV vaccine; to our knowledge, this also is the first isolation of human MAbs from an individual immunized with an inactivated flavivirus vaccine. We identified two strongly neutralizing JEV-specific human MAbs: one (hJEV-69) that recognized E-DIII-LR and another (hJEV-75) that mapped to residues in the E-DI-LR, E-DI-DII-hinge, E-DII-LR, and E-DII-hinge. Future studies will need to assess the inhibitory potential of the anti-JEV humoral response against contemporary strains of JEV of all genotypes, including GV strains.

Type-specific and cross-reactive neutralizing MAbs have been identified against JEV. Although others have identified E-DIII-specific anti-JEV MAbs from mice (25, 27, 28), this class of antibodies appears less immunodominant in humans, at least against some (40–44) but not all (45, 46) flaviviruses. Murine-derived E-DIII-specific MAbs (2H4, A3, and E3.3) against JEV had stronger neutralizing activity *in vitro* than E-DII-specific MAbs (25, 30, 47, 48). Humanization of chimpanzee-derived E-DI (A3 and B2)- and E-DIII (E3)-specific MAbs demonstrated equivalent *in vitro* neutralization compared to the parental MAbs, and this finding correlated with protection against JEV infection in mice by the homologous genotype (GIII) (29).

We performed epitope-mapping studies on our mouse MAbs by using complementary approaches: HDX-MS and alanine-scanning mutagenesis. Epitope mapping by HDX-MS identified a series of short peptides that were recognized by our strongest neutralizing E-DIII-specific MAbs (JEV-31, JEV-128, JEV-131, and JEV-143). Subsequent analysis by alanine-scanning mutagenesis confirmed and extended these findings by defining individual amino acid residues in E-DIII-LR (T321, D332, and I383) required for optimal MAb binding (JEV-31, JEV-131, JEV-143, and hJEV-69). HDX provided information on MAb reactivity with a peptide segment but lacked residue-level specificity. Reciprocally, alanine-scanning mutagenesis defined specific amino acids required for optimal binding but is of limited utility if mutation of more than one residue is required for significant loss of binding. Loss-of-binding analysis of the neutralizing hJEV-75 MAb identified residues across E-DI and E-DII, particularly within the previously defined E-DI-LR, E-DII-LR, and E-DI-DII-hinge epitopes. JEV-117, a mouse MAb that was poorly neutralizing, exhibited a similar loss-of-binding profile to hJEV-75. Although further studies are warranted, the differential functional activities of JEV-117 and hJEV-75 may be due to differences in accessibility of their epitopes or affinity of binding. Higher-resolution studies, including X-ray crystallography and cryo-electron microscopy, are necessary to determine the precise geometry of binding and a complete footprint of interacting residues.

We observed some variation in neutralizing activity of some MAbs against different JEV strains and genotypes. This piece of data is analogous to that observed with MAbs against different DENV-3 genotypes (49, 50). The intergenotypic amino acid sequence divergence in the E protein among genotypes ranges from 0.6% (GII versus GIII) to 5.6% (GIII versus GIV) (51). Infection with one JEV genotype is believed to confer long-term immunity against both homologous and heterologous genotypes. We assumed it might be straightforward to generate mouse and human MAbs that neutralized all JEV genotypes available to us. Indeed, there are limited amino acid changes in E-DIII among the JEV strains that we tested, with only 5 amino acid differences (residues 315, 327, 333, 336, and 366); accordingly, the variation in neutralization of different JEV genotypes by E-DIII-specific MAbs was limited (<10-fold). Two MAbs (JEV-117 and hJEV-75) effectively neutralized the JEV-SA14-14-2 vaccine strain but remarkably lost inhibitory activity against the parental JEV-SA14 strain. These MAbs mapped to epitopes that also contained residues outside E-DIII, in E-DI and E-DII. An alignment of the genotypic variation in JEV sequences (Fig. S3) failed to show a direct correlation with the residues identified in loss-of-binding studies for JEV-117 and hJEV-75. Although the sites of genotypic variation between JEV-SA14-14-2 and JEV-SA14 are not coincident with JEV-117 or hJEV-75 epitope residues, there are several residues in close proximity. For JEV-117, the H/Q264 genotypic variation is within 5 Å of the epitope residue at position 262; M/K279 also is within 5 Å of epitope residue 49, and the K/E138 site of genotypic variation is within 10 Å of epitope residue 136. For hJEV-75, the M/K279 genotypic variation is within 5 Å of epitope residue 49 or within 10 Å of epitope residues 273 and 275. Similarly, the K/E138 site of genotypic variation is within 10 Å of epitope residue 49, and the H/Q264 site of genotypic variation is also within 10 Å of the epitope residue 262. As an alternative explanation, differences in strain and genotype residues allosterically could affect the display of JEV-117 and hJEV-75 epitopes. This idea has been described as a basis for differential neutralization of flavivirus genotypes by other antibodies (52, 53). Clearly, further studies with higher-resolution epitope mapping of the JEV-117 and hJEV-75 MAbs (e.g., atomic resolution structures of the Fab-E complexes) may resolve this question of differential neutralization of JEV strains. Overall, our results have potential implications for assessing the breadth of the protective efficacy of existing and new JEV vaccines. It may be critical to assess whether antibody responses against the vaccine strain of a given JEV efficiently neutralize infection of heterologous genotypes that may emerge.

10.1128/mBio.00008-18.3 FIG S3  Alignment of sequences of different JEV strains. Genotypic differences from the JEV-SA14-14-2 strain (GIII) are highlighted as shaded black residues. The secondary structure elements above the alignment are derived from the structure of JEV E protein (PDB accession no. 3P54). The GenBank identification numbers for each viral sequence are described in Materials and Methods. The JEV-Nakayama strain includes an unknown amino acid at position 209 (denoted by an X). Download FIG S3, TIF file, 4.3 MB. Copyright © 2018 Fernandez et al. 2018 Fernandez et al. This content is distributed under the terms of the Creative Commons Attribution 4.0 International license.

Mechanism-of-action studies showed that all neutralizing murine E-DIII-LR MAbs could block virus fusion, as was observed previously for E16, a WNV-specific MAb (33). Although hJEV-69 exhibited a loss-of-binding profile similar to those of E-DIII-LR-specific mouse MAbs, charge substitutions in this region (S331K and S364K) did not affect hJEV-69 binding, suggesting a somewhat unique epitope. Consistent with this observation, FFWO studies of hJEV-69 indicated that although it inhibited at a postattachment stage, it did not efficiently block pH-dependent fusion. Although further studies are required, the neutralizing human MAbs could block at a postentry step before fusion. Alternatively, the FFWO, which is a measure of viral fusion at the plasma membrane, may not fully recapitulate the events occurring in the late endosome.

We performed protection studies *in vivo* with our mouse and human MAbs and JEV strains corresponding to the two most commonly circulating genotypes (GI and GIII). To our knowledge, the protective effect of JEV MAbs against genotype I strains *in vivo* has not been studied previously. Several of our neutralizing MAbs (JEV-31, JEV-106, JEV-131, JEV143, JEV-169, and hJEV-75) completely protected against lethal JEV-Nakayama (GIII) infection when administered as prophylaxis. A subgroup of MAbs (JEV-31, JEV-131, and JEV-169) also completely protected against JEV-2372/79, a GI strain, with all MAbs tested partially preventing lethal infection by a highly homologous second GI strain, JEV-MAR 859, with 99% amino acid identity at the E protein. Remarkably, postexposure therapeutic administration of a single dose of JEV-31 or JEV-169 at 5 days after infection also conferred complete or partial protection against GIII or GI strains, respectively. A single postexposure dose of hJEV-75 also provided high levels of protection against GI or GIII strains. Although prior studies have reported *in vivo* efficacy of murine and humanized E-DIII MAbs against JEV (26, 29, 30), these challenge studies were performed with single, homologous JEV genotypes, and protection was limited to prophylaxis, with the exception of a single study (30). The postexposure protection we observed is similar to that seen previously for other E-DIII-LR MAbs, including E16 and WNV (54) and E106 and DENV-1 (55). One caveat of our study is that administration of anti-JEV antibody at day 5 preceded the development of central nervous system symptoms (e.g., seizures, tremors, paralysis, or lethargy). More detailed window-of-treatment analysis is needed to determine which MAbs retain protective efficacy after the development of disease onset.

In summary, we identified a panel of anti-JEV MAbs that map to epitopes in E-DI and E-DIII with broadly neutralizing activity against multiple JEV genotypes. Although both mouse and human neutralizing MAbs can block infection at a postattachment stage, the mouse MAbs appear to have a greater capacity to block pH-dependent viral fusion. Studies using liposome-based fusion experiments (32, 33, 56) and cell entry assays (33) will be required to corroborate these findings. Overall, our combination of *in vitro* MAb neutralization analyses with mechanism of action, epitope mapping, and *in vivo* activity provides insight into developing and refining vaccine and therapeutic countermeasures against emerging JEV strains and genotypes.

## MATERIALS AND METHODS

### Viruses.

JEV strains 2372/79 (Thailand 1979, GenBank accession no. U70401), MAR 859 (Cambodia 1967, accession no. U70410), Bennett (Korea 1951, accession no. HQ223285), Nakayama (Japan 1935, accession no. EF571853), SA14-14-2 (China 1954, accession no. JN604986), SA14 (China 1954, accession no. M55506), and JKT 7887 (Indonesia 1981; accession no. L42160) were provided by the World Reference Center for Emerging Viruses and Arboviruses (K. Plante, S. Weaver, and R. Tesh, Galveston, TX). Virus stocks were propagated in C6/36 *Aedes albopictus* cells for 5 days prior to collection, and their titers were determined by focus-forming assay (FFA) on Vero cell monolayers, as described previously (57).

### MAb generation. (i) Mouse MAbs.

*Irf3*^*−/−*^ mice were infected and boosted with 10^2^ FFU of JEV-SA14-14-2 and given a final intravenous boost with 10^6^ FFU of JEV-SA14-14-2 3 days prior to fusion with P3X63.Ag.6.5.3 myeloma cells. *Irf7*^*−/−*^ mice were infected and boosted with 10^2^ FFU of JEV-Nakayama and JEV-Bennett, respectively, and given a final boost with 10^3^ FFU of JEV-Nakayama 3 days prior to fusion. Antibodies from hybridomas that bound to JEV-infected Vero cells by flow cytometry and JEV-SA14-14-2 by direct ELISA were cloned by limiting dilution. All hybridomas were screened initially with a single-endpoint neutralization assay using neat hybridoma supernatant incubated with 10^2^ FFU of JEV-SA14-14-2 for 1 h at 37°C. MAb-virus complexes were added to Vero cell monolayers for 1 h at 37°C followed by 1% (wt/vol) methylcellulose in modified Eagle medium (MEM) supplemented with 4% fetal bovine serum (FBS). Plates were fixed with 2% paraformaldehyde (PFA) in phosphate-buffered saline (PBS) 30 h later and sequentially stained with 500 ng/ml WNV E60 (cross-reactive MAb) (38) and horseradish peroxidase (HRP)-conjugated goat anti-mouse IgG in PBS supplemented with 0.1% saponin and 0.02% Tween 20. JEV-infected foci were visualized using TrueBlue peroxidase substrate (KPL) and quantitated on an ImmunoSpot 5.0.37 macroanalyzer (Cellular Technologies). Hybridoma supernatants with greater than 85% neutralization were purified commercially (Bio-X Cell) after adaptation for growth under serum-free conditions.

### (ii) Human MAbs.

The human donors used in this study were born in the United States and Colombia and had not experienced prior JEV infection. However, they were not tested for prior exposure to other flaviviruses (e.g., WNV or DENV). Donors were immunized voluntarily with a two-dose regimen of a commercially available inactivated JEV vaccine, IXIARO, as part of an occupational exposure program. Peripheral blood was obtained for research purposes after informed consent approximately 1 month after boosting, with prior Institutional Review Board approval from Vanderbilt University Medical Center. Peripheral blood mononuclear cells (PBMCs) from heparinized blood were isolated using Ficoll-Histopaque and density gradient centrifugation. The cells were cryopreserved in the vapor phase of liquid nitrogen until use. Ten million PBMCs were cultured in 384-well plates (Nunc) using culture medium (ClonaCell-HY medium A; StemCell Technologies) supplemented with 8 μg ml^−1^ of the Toll-like receptor (TLR) agonist CpG (phosphorothioate-modified oligodeoxynucleotide ZOEZOEZZZZZOEEZOEZZZT; Invitrogen), 3 μg ml^−1^ of Chk2 inhibitor (Sigma), 1 μg ml^−1^ of cyclosporine (Sigma), and clarified supernatants from cultures of B95.8 cells (ATCC) containing Epstein-Barr virus. After 7 days, cells from each 384-well culture plate were expanded into four 96-well culture plates (Falcon) using ClonaCell-HY medium A containing 8 μg ml^−1^ of CpG, 3 μg ml^−1^ of Chk2 inhibitor, and 10^7^ irradiated heterologous human PBMCs (Nashville Red Cross) and cultured for an additional 4 days. Supernatants were screened by ELISA (described below) for reactivity with JEV-SA14-14-2. Hybridoma cell lines were cloned by single-cell flow cytometric sorting in a sterile FACSAria III cytometer (BD Biosciences).

### Neutralization assays.

Serial dilutions of MAbs were incubated with 10^2^ FFU of different JEV strains for 1 h at 37°C as described previously (57). MAb-virus complexes were added to Vero cell monolayers for 1 h at 37°C followed by 1% (wt/vol) methylcellulose in modified Eagle medium (MEM) supplemented with 4% FBS. Plates were fixed and processed as described above. Nonlinear regression analysis was performed, and EC_50_ values were calculated after comparison to wells infected with JEV in the absence of MAb.

### Flavivirus E ectodomain and JEV E-DI and JEV E-DIII expression and purification.

JEV E protein (residues 1 to 399 corresponding to the E ectodomain of the JEV-SA14-14-2 strain) was prepared as previously described (15). A JEV E-DI synthetic gene was designed based on a DENV-4 DI construct (58) with modifications such that JEV E residues 1 to 50 were linked to residues 135 to 195 by a glycine dipeptide, and residues 135 to 195 were connected by a serine residue to residues 281 to 298. This fragment was cloned into the pFM1.2 mammalian expression vector (59) downstream of a pHLsec signal sequence and terminated with a C-terminal tobacco etch virus (TEV) protease and hexahistidine affinity tag. Transient expression and purification were completed using established protocols (60). JEV E-DIII (residues 299 to 399) was cloned into the NdeI and XhoI restriction enzyme sites of pET21a for expression in BL21(DE3) Codon Plus *Escherichia coli* cells by autoinduction (61). The protein was refolded from inclusion bodies and purified by size exclusion essentially as described previously (62). WNV (63) and ZIKV (60) E ectodomain proteins were produced and purified based on established protocols.

### JEV MAb domain mapping.

MaxiSorp 96-well plates (Thermo, Fisher) were coated with 50 μl of 4 μg/ml of recombinant JEV E (15), JEV E-DI, JEV E-DIII, WNV E, or ZIKV E overnight at 4°C. Plates were washed three times with PBS with 0.02% Tween 20 followed by incubation with PBS and 2% bovine serum albumin (BSA) for 1 h at 37°C. MAbs were added (1 μg/ml) for 1 h at room temperature. Plates were washed again and sequentially incubated with biotin-conjugated anti-mouse IgG, streptavidin-HRP, and 3,3′,5,5′-tetramethylbenzidine (TMB) substrate. The reaction was stopped by addition of 2 M H_2_SO_4_, and emission (450 nm) was read using a TriStar LB 941 reader (Berthold Technologies).

### Pre- and postattachment neutralization assays.

For preattachment assays, serial dilutions of MAbs were prepared at 4°C in Dulbecco’s modified Eagle medium (DMEM) with 2% FBS and preincubated with 10^2^ FFU of JEV-SA14-14-2 for 1 h at 4°C. MAb-virus complexes were added to a monolayer of Vero cells for 1 h at 4°C. Unbound virus was removed with three washes of chilled DMEM, and adsorbed virus was allowed to internalize during a 37°C incubation for 1 h. Cells were overlaid with 1% (wt/vol) methylcellulose in MEM supplemented with 4% FBS. For postattachment assays, 10^2^ FFU of JEV-SA14-14-2 was adsorbed onto a monolayer of Vero cells for 1 h at 4°C. After removal of unbound virus, serial dilutions of MAbs were added to virus-adsorbed cells for 1 h at 4°C. Virus then was allowed to internalize for 1 h at 37°C, and subsequently cells were overlaid with methylcellulose as described above. Thirty hours later, the plates were fixed with 2% PFA and analyzed for antigen-specific foci as described above.

### Fusion blockade assay.

The assay for plasma membrane fusion inhibition with flavivirus MAbs was described previously (32–34). Briefly, Vero cells (2 × 10^4^ per well) were seeded in flat-bottom 96-well plates overnight at 37°C. The following day, cells were preincubated with 10 nM concanamycin A (Sigma catalog no. C9705), which blocks acidification of endosomes and viral fusion, for 30 min on ice and subsequently incubated with JEV-SA14 (multiplicity of infection [MOI] of 50) for 2 h. Cells were washed twice with chilled PBS followed by incubation with 100 μg/ml (murine) or 50 μg/ml (human) MAbs for 30 min on ice. Cells were pH shifted with warmed DMEM (buffered to pH 5.5 or control pH 7.5) at 37°C for ~7 min. The cells were rinsed and incubated for 24 h at 37°C in DMEM with 10 nM concanamycin A. Subsequently, cells were rinsed, fixed, permeabilized, and sequentially stained for 1 h at 4°C with JEV-13 (1 μg/ml) and goat anti-mouse Alexa Fluor 647-conjugated secondary antibody (1:2,000). Samples were processed by flow cytometry (MacsQuant), and data were analyzed using FlowJo software.

### Hydrogen-deuterium exchange.

Continuous HDX labeling of JEV E-DIII with or without the MAbs was performed at 25°C for 0, 10, 30, 60, 120, 900, 3,600, and 14,400 s as previously described with the following modifications (64). Briefly, stock solutions of JEV E-DIII both with and without the MAbs were prepared in PBS (pH 7.4) and incubated at 25°C for at least 1 h. Continuous labeling with deuterium was initiated by diluting the stock samples 10-fold in deuterated PBS buﬀer (Sigma-Aldrich). HDX control samples (nondeuterated) were prepared in the same way with H_2_O. Quenching was performed under reducing conditions by adding a solution of 500 mM Tris (2-carboxyethyl)phosphine hydrochloride (TCEP HCl) and 4 M guanidine hydrochloride in PBS buffer (pH 7.4 [adjusted using sodium hydroxide]) to the reaction vial at a 1:1 vol/vol ratio. The sample was mixed and incubated for a minute at 25°C before being loaded onto our custom-built HDX platform for desalting, online pepsin digestion, and reversed-phase separation and directly injected into the mass spectrometer for analysis.

The sample was passed over a custom-packed 2- by 20-mm pepsin column at 200 µl/min; immobilized pepsin was prepared according to a published protocol (65). The peptides resulting from digestion were captured by a 2.1- by 20-mm Zorbax Eclipse XDB-C_8_ trap column (Agilent) and desalted at 200 µl min^−1^ of H_2_O containing 0.1% trifluoroacetic acid for 3 min. The peptides were separated by a 2.1 × 50 mm C_18_ column (2.5-µm XSelect CSH C_18_; Waters) with a 9.5-min gradient of 5 to 100% acetonitrile in 0.1% formic acid at a flow rate of 100 μl min^−1^ delivered by a LEAP 3× Ti pump (Leap Technologies, NC). The linear part of the gradient from 0.3 min to 5.5 min raised the acetonitrile content from 15% to 50%, during which time most of the peptides eluted from the C_18_ column. The entire fluidic system was kept in an ice bath, except for the pepsin column, to minimize back exchange. Duplicate measurements were carried out for each of the time points.

### HDX data analysis and epitope assignment.

Acquired spectra were analyzed using HDX workbench software (66) against a peptide set generated as described below. The deuterium level was normalized to the maximum deuterium concentration (80%) contained in the reaction vial. The peptide list used to search the HDX data was identified first by a tandem-MS experiment in a data-dependent mode on a linear trap quadrupole-Fourier transform (LTQ-FT) mass spectrometer (Thermo). The six most abundant ions were submitted to collision-induced dissociation fragmentation. Product-ion spectra were then submitted to MassMatrix (version 2.4.2) for identification (67) and manually inspected, and the validated peptides were used for the HDX analysis. The epitopes were identified as regions/sequences of amino acids (not single residues) that show a significant difference in HDX for the bound versus unbound states, as determined from the peptide-level HDX-MS data. Criteria for the selection of peptides as potential epitopes are explained further in the Immune Epitope Database (IEDB) submission mentioned below in the “Accession number(s)” section.

### Site-directed mutagenesis epitope mapping.

Epitope mapping was performed by alanine-scanning mutagenesis as described previously (36). A JEV prM-E protein expression construct (based on JEV-SA14-14-2) was subjected to commercial alanine-scanning mutagenesis (Genewiz) to generate a mutant library. Each residue within the JEV E protein was changed to alanine, with alanine codons mutated to serine and cysteine residues left unchanged. In total, 400 mutants were generated and sequence confirmed. Each JEV E protein mutant was transfected into human 293T cells and allowed to express for 24 h and then fixed and permeabilized with Foxp3 transcription factor staining buffer (Thermo catalog no. 00-5523-00). Cells were incubated sequentially with purified MAbs at concentrations optimized for staining (range, 30 to 1,000 ng/ml) and Alexa Fluor 647-conjugated secondary antibody (Invitrogen) in permeabilization buffer. Fluorescence signal was detected by flow cytometry (MacsQuant) and analyzed using FlowJo software. Antibody reactivity against each mutant was compared to that of the WT prM-E protein after subtracting the signal from mock-transfected controls and normalizing to the signal from WT prM-E transfected controls. Mutations were identified as critical to the MAb epitope if the mutants showed less than 25% binding compared to the wild type. For charge mutants, we substituted residues in the A strand (S309K, K312E, and H395K), DIII-LR (S331K, S364K, N367K, and K369E), C-C′ loop (T349K), and FG loop (R387E and D389K) and transfected and stained as described above.

### Mouse experiments.

Animal studies were carried in accordance with the recommendations of the *Guide for the Care and Use of Laboratory Animals* of the National Institutes of Health and were approved by the Institutional Animal Care and Use Committee at the Washington University School of Medicine (assurance no. A3381-01). Mice were inoculated with JEV after induction of anesthesia using ketamine hydrochloride and xylazine, and all efforts were made to minimize pain and suffering. Antibody protection studies were performed according to the models described below.

### (i) Genotype I.

WT C57BL/6 male mice (3 weeks old; Jackson Laboratories) were inoculated with 10^3^ FFU of JEV-MAR 859 or JEV-2372/79 subcutaneously in the footpad. Anti-JEV or isotype control (CHK-152) MAbs were administered intraperitoneally as a single dose on day −1 (10 μg, 0.5 mg/kg) or day 5 (250 μg, 12.5 mg/kg) after infection. Animals were monitored for lethality for 28 days.

### (ii) Genotype III.

WT C57BL/6 male mice (4 to 5 weeks old; Jackson Laboratories) were inoculated with 10^2^ FFU of JEV-Nakayama subcutaneously in the footpad. Anti-JEV or isotype control (CHK-152) MAbs were administered intraperitoneally as a single dose on day −1 (10 μg, 0.5 mg/kg) or day 5 (250 μg, 12.5 mg/kg) after infection. Animals were monitored for lethality for 28 days.

### Statistical analysis.

Statistical significance of FFWO was determined by one-way ANOVA with Dunnett’s multiple comparisons to an isotype control MAb. Statistical significance of alanine shotgun mutagenesis was determined by one-way ANOVA with Holm-Sidak’s multiple comparisons of each mutant to V315 for each MAb. Kaplan-Meier survival curves were analyzed by the log rank test for each MAb compared to an isotype control MAb.

### Accession number(s).

The epitopes of the five JEV-specific MAbs (E31, E106, E128, E131, and E143) have been deposited in the Immune Epitope Database (IEDB) under submission no. 1000721.
