# Supplementary figures and images for: Mouse and Human Monoclonal Antibodies Protect against Infection by Multiple Genotypes of Japanese Encephalitis Virus
Source: mBio. 2018 Feb 27;9(1):e00008-18. doi: 10.1128/mBio.00008-18 (PMC5829823; doi:10.1128/mBio.00008-18)

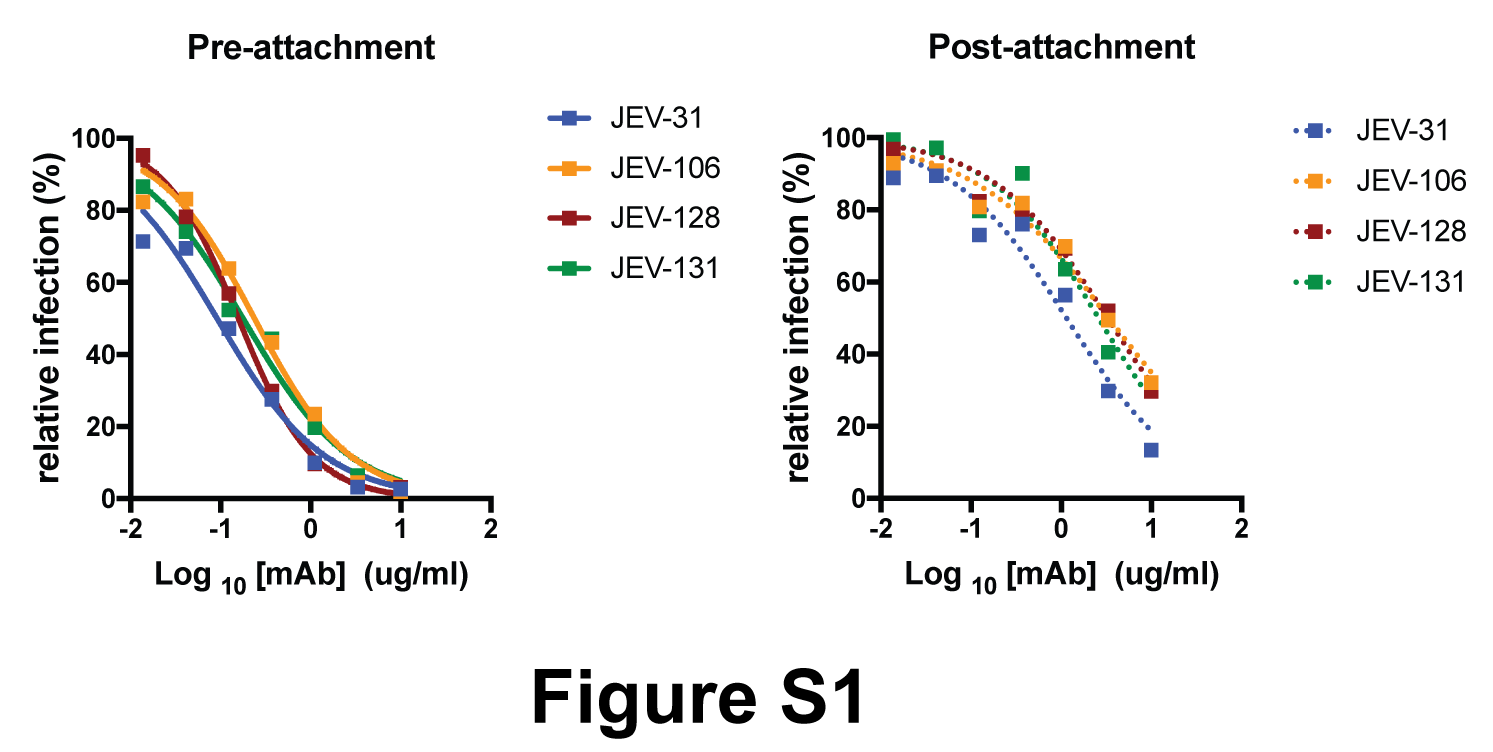

Supplement: FIG S1 [file mbo001183744sf1.tif]

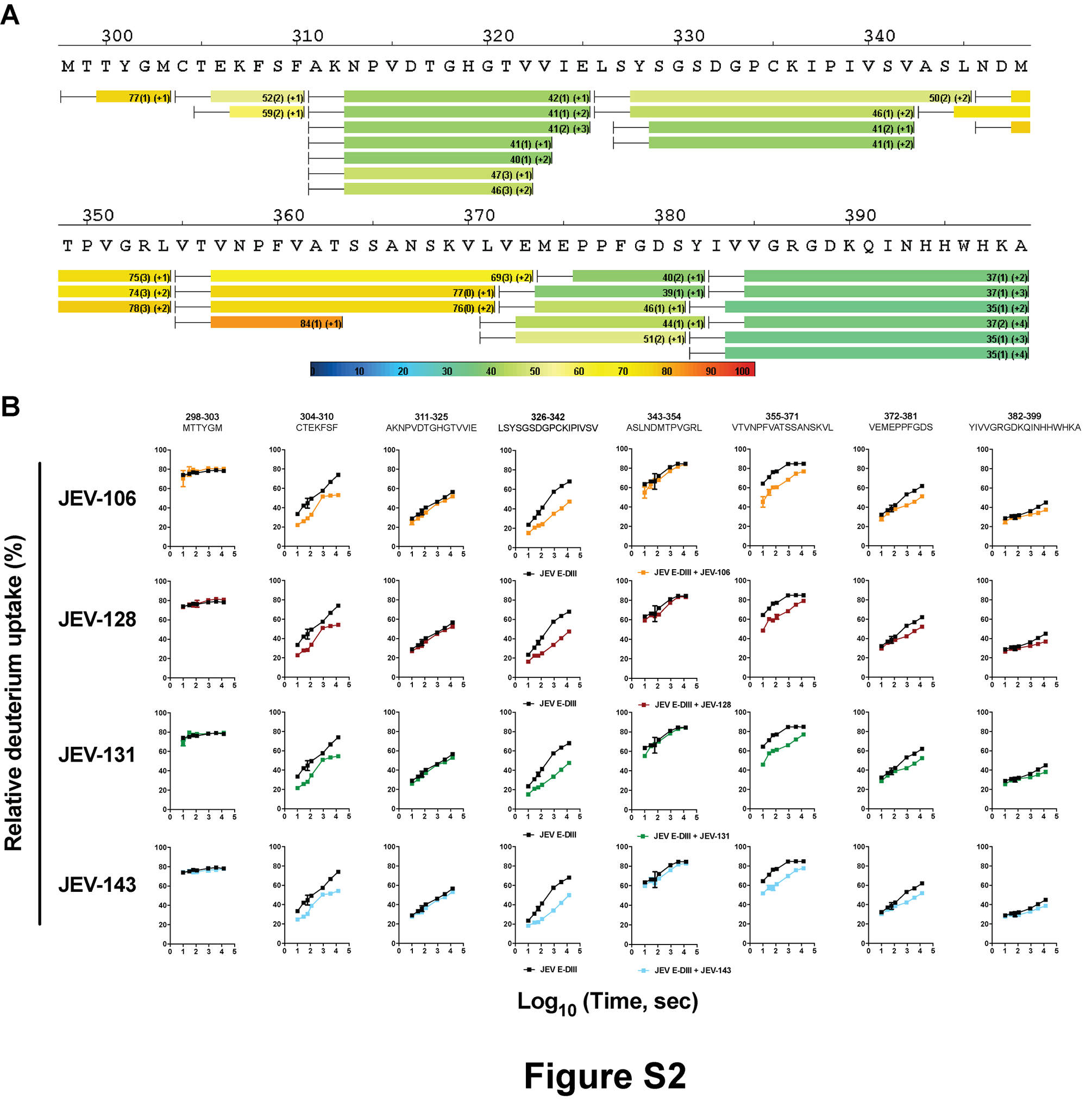

Supplement: FIG S2 [file mbo001183744sf2.tif]

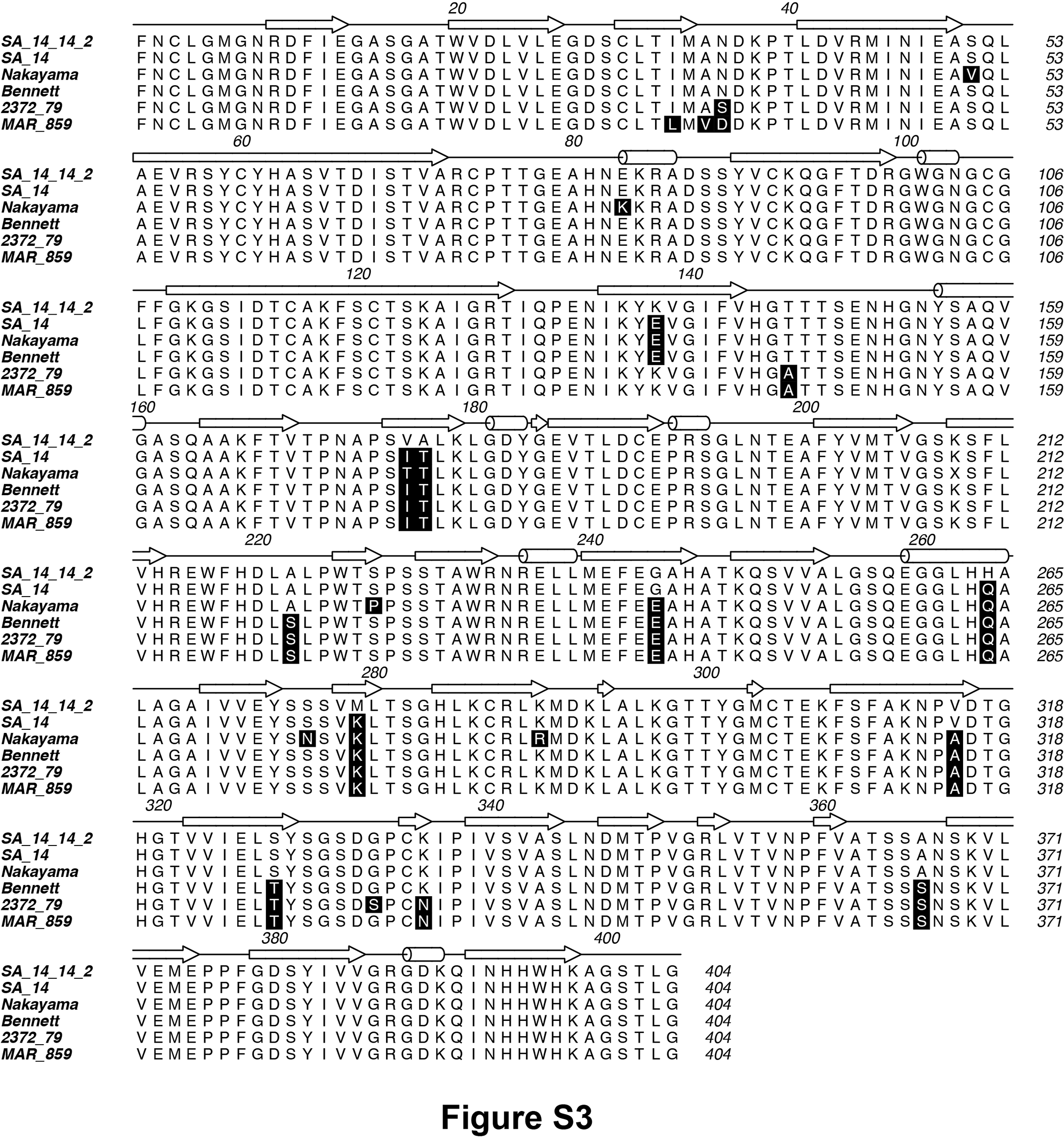

Supplement: FIG S3 [file mbo001183744sf3.tif]
